# Genome-wide analysis of soybean hypoxia inducible gene domain containing genes: a functional investigation of GmHIGD3

**DOI:** 10.3389/fpls.2024.1403841

**Published:** 2024-07-01

**Authors:** Xiaoyan Geng, Lu Dong, Tiantian Zhu, Chunhong Yang, Jianhua Zhang, Binhui Guo, Huatao Chen, Qun Zhang, Li Song

**Affiliations:** ^1^ Joint International Research Laboratory of Agriculture and Agri-Product Safety, the Ministry of Education of China, Jiangsu Key Laboratory of Crop Genomics and Molecular Breeding, Yangzhou University, Yangzhou, Jiangsu, China; ^2^ Zhongshan Biological Breeding Laboratory, Nanjing, China; ^3^ Institute of Industrial Crops, Jiangsu Academy of Agricultural Sciences, Nanjing, China; ^4^ College of Life Sciences, State Key Laboratory of Crop Genetics & Germplasm Enhancement and Utilization, Nanjing Agricultural University, Nanjing, China

**Keywords:** HIGD, *Glycine max*, expression pattern, hypoxia response, mitochondria

## Abstract

The response of Hypoxia Inducible Gene Domain (HIGD) proteins to hypoxia plays a crucial role in plant development. However, the research on this gene family in soybean has been lacking. In this study, we aimed to identify and comprehensively analyze soybean *HIGD* genes using the *Glycine max* genome database. As a result, six *GmHIGD* genes were successfully identified, and their phylogeny, gene structures, and putative conserved motifs were analyzed in comparison to *Arabidopsis* and rice. Collinearity analysis indicated that the *HIGD* gene family in soybean has expanded to some extent when compared to *Arabidopsis*. Additionally, the cis-elements in the promoter regions of *GmHIGD* and the transcription factors potentially binding to these regions were identified. All *GmHIGD* genes showed specific responsiveness to submergence and hypoxic stresses. Expression profiling through quantitative real-time PCR revealed that these genes were significantly induced by PEG treatment in root tissue. Co-expressed genes of *GmHIGD* were primarily associated with oxidoreductase and dioxygenase activities, as well as peroxisome function. Notably, one of *GmHIGD* genes, GmHIGD3 was found to be predominantly localized in mitochondria, and its overexpression in *Arabidopsis* led to a significantly reduction in catalase activity compared to wild-type plants. These results bring new insights into the functional role of GmHIGD in terms of subcellular localization and the regulation of oxidoreductase activity.

## 1 Introduction

Soybean provides humans with a large amount of protein, essential amino acids, oil, and metabolizable energy (León et al., 2021; Zhou et al., 2021). However, soybean is extremely sensitive to flooding stress during growth and development. Flooding stress can significantly affect growth, grain yield, and seed quality by reducing plant growth, nitrogen fixation, photosynthesis, biomass accumulation, stomatal conductance, and nutrition availability from soil (Githiri et al., 2006; Valliyodan et al., 2017; Ye et al., 2018). Flooding stress can occur in two forms: submergence stress, where the plant organ is completely under water, and waterlogging stress, where the plant’s leaves and stems are partially submerged (Nishiuchi et al., 2012). It was estimated that waterlogging can reduce soybean yield by 17−43% during the vegetative growth stage and 50−56% during the reproductive stage (Oosterhuis et al., 1990). Even just two days of waterlogging can reduce soybean production by 27% (Linkemer et al., 1998). Additionally, flooding can increase the risk of plant pathogens and the occurrence of crop disease after floods (Gravot et al., 2016).

The primary and most direct impact of flooding stress is oxygen deprivation. In flooded conditions, the lack of oxygen (O_2_) in the water can cause cellular damage and then restrain plant growth. This, in turn, prevents the production of glucose, leading to various metabolic issues. The severity of these negative effects increases with prolonged submergence and high temperatures, which elevate oxygen consumption through plant respiration (Deutsch et al., 2015). Additionally, certain developmental stages of plants, such as seed germination, early growth after germination, and flowering, are particularly sensitive to low O_2_ availability (Considine et al., 2017; Le Gac and Laux, 2019). Previous studies have shown that soybean plants are most vulnerable to flooding damage during early growth stages when secondary aerenchyma in roots has not yet formed and could not provide an oxygen pathway under flooded conditions (Shimamura et al., 2003; Valliyodan et al., 2017).

Hypoxia Inducible Gene Domain (HIGD) protein family in mammals consists of five homologs, namely *HIGD−1A*, *HIGD−1B*, *HIGD−1C*, *HIGD−2A* and *HIGD−2B* (Bedo et al., 2004). HIGD1A, initially identified and studied in humans, is a mitochondrial inner membrane protein of approximately 10 kDa that is induced by hypoxia-inducible factor 1 (HIF-1). It interacts with the mitochondrial electron transport chain to reduce oxygen consumption and plays a role in both cell death and survival, depending on the developmental stage and cellular microenvironment (Hayashi et al., 2012; Ameri et al., 2013). It has been reported that HIGD1B acts as an inhibitor to prevent hypoxia−induced mitochondrial fragmentation (Pang et al., 2021). HIGD1C is essential for oxygen sensing in the carotid body and increases the sensitivity of complex IV to hypoxia (Timón-Gómez et al., 2022).

The *HIGD* gene family has been identified in both *Arabidopsis* and rice plants. In rice, there are five *HIGD* genes: *OsHIGD2*, *OsHIGD3* and *OsHIGD5* respond to submergence, hypoxia, and ethylene at different time points, while *OsHIGD1* and *OsHIGD4* exhibit almost undetectable expression under all conditions (Hwang and Choi, 2016). In *Arabidopsis*, *AtHIGD1* expression levels are induced by hypoxia treatment, and overexpression of the *AtHIGD1* gene has been shown to enhance survival rates after hypoxia stress compared to wild-type plants (Hwang et al., 2017). Both OsHIGD2 and AtHIGD1 localize to mitochondria (Hwang and Choi, 2016; Hwang et al., 2017). However, no information is available on the hypoxia response information of the soybean *HIGD* family. During the onset of hypoxia-triggered responses, responsive genes are expected to play key regulatory roles and the detrimental effects of hypoxia can sometimes be counteracted through the induced expression of genes encoding proteins that promote immunity (Hsu et al., 2013). To better understand soybean adaptation to flooding stress and hypoxia environments, we characterized the soybean *HIGD* gene family in terms of sequence, structure, phylogenetic relationships, gene structure, conserved motifs, and chromosomal localization. We also analyzed their expression levels in various tissues and under different stress conditions. Overall, our results provide detailed insights into the soybean *HIGD* family and could facilitate a more comprehensive understanding of the function of *HIGD* genes in soybean.

## 2 Materials and methods

### 2.1 Identification of *GmHIGD* genes from the *G.max* genomic sequence and phylogenetic analysis

Soybean predicted proteins were obtained from the Phytozome database (https://phytozome-next.jgi.doe.gov/, v13) (Goodstein et al., 2012). GmHIGD proteins were identified using BLASTp searches against the soybean predicted proteins, with *Oryza sativa* and *Arabidopsis* HIGD proteins as queries. All potential GmHIGD sequences were analyzed to verify the presence of the conserved hypoxia induced protein region (HIG_1_N, PF04588) using Pfam tools (https://pfam.xfam.org/). Sequences lacking the conserved regions were manually removed. A neighbor-joining (NJ) tree was constructed using MEGA 7.0 with 1000 bootstrap replicates, including HIGD proteins from rice and *Arabidopsis*. Gene names from GmHIGD1 to GmHIGD6 were assigned according to their positions in the phylogenetic tree. All protein amino acid sequences that used for phylogenetic analysis were provided in **Supplementary File 1**.

### 2.2 Gene structure and conserved motif analysis

Gene structure information was retrieved from the *Glycine max* genome data, and visualized using TBtools software (Chen et al., 2020). The exon-intron structures of *GmHIGD* genes were analyzed by aligning the coding sequences with their corresponding genomic sequences and visualized using the online software GSDS (http://gsds.gao-lab.org/index.php, Hu et al., 2015). The amino acid sequences of GmHIGDs were analyzed using the MEME tool (http://meme-suite.org/index.html) to identify conserved domains and motifs within each group. The analysis included setting the maximum number of motifs to 5, with a minimum width of 6 and a maximum width of 50 amino acid residues, and an e-value threshold of less than 1x10-^8^.

### 2.3 Protein properties, 3-D domain and subcellular localization

The physical and chemical properties of GmHIGD proteins were analyzed using the ProtParam online tool (https://web.expasy.org/protparam/, Gasteiger et al., 2005). Subcellular localization predictions for these GmHIGD proteins were carried out using various tools including the CELLO v.2.5: subCELlular LOcalization predictor (http://cello.life.nctu.edu.tw/) (Yu et al., 2006), WoLF_PSORT tool (http://www.genscript.com/wolfpsort.html), the mGOASVM server (http://bioinfo.eie.polyu.edu.hk/mGoaSvmServer/mGOASVM.html, Plant V2), and the Plant-mPLoc database (http://www.csbio.sjtu.edu.cn/bioinf/plant-multi, Chou and Shen, 2010; Wan et al., 2012). Additionally, the Phyre2 server (http://www.sbg.bio.ic.ac.uk/phyre2/html/page.cgi?id=index) was used for homology modelling to predict the three-dimensional (3D) structure of HIGD proteins from soybean, *Arabidopsis*, and rice.

### 2.4 Chromosomal location analysis and gene duplication

The chromosomal locations of *GmHIGD* genes were determined based on the *Glycine max* genome annotation and visualized using TBtools software (https://github.com/CJ-Chen/TBtools-Manua, Chen et al., 2020). Duplication events in *GmHIGD* genes within the soybean genome were detected using the Multiple Collinearity Scan toolkit (MCScanX) and visualized with the CIRCOS program (Krzywinski et al., 2009; Wang et al., 2012).

### 2.5 Putative cis-elements in the promoter regions

The 2,000 bp sequences upstream from the translation start codon of all *GmHIGD* genes were obtained from Phytozome v13. Putative cis-acting regulatory elements within these sequences were predicted using the PlantCARE online database (http://bioinformatics.psb.ugent.be/webtools/plantcare/html/, accessed on 22 Feb 2022) (Lescot et al., 2002) and the PLACE website (https://www.dna.affrc.go.jp/PLACE/?action=newplace).

### 2.6 Co-expression and association genes of GmHIGD analysis

Association genes of *GmHIGDs* were downloaded from the STRING database, which provides functional protein association networks (https://cn.string-db.org, v11.5; Szklarczyk et al., 2021). Co-expression-based gene network analysis was performed on all *GmHIGD* genes using Spearman correlation coefficients to identify relevant genes from RNA-Seq data. Gene selection was based on a co-expression value greater than 0.7. All of these associated genes and co-expressed genes were subjected to Kyoto Encyclopedia of Genes and Genomes (KEGG) enrichment analysis.

### 2.7 Subcellular location of GmHIGD3

Firstly, the GmHIGD3 coding sequence without the stop codon was cloned into vector pHB-35S-mCherry to generate C-terminal mCherry fusions. Then, the obtained pHB-GmHIGD3-mCherry fusion plasmid was transformed into *Escherichia coli* DH5α and verified by sequencing (Sangon, Shanghai, China). Co-expression of the plasmids pK7FWG2-REM1.2-EGFP (membrane protein marker, Huang et al., 2019), pCAMBIA1301-EGFP-AtCAT2 (peroxisome protein marker), pCAMBIA1301-GFP-GLP_151_-P2P3 (plastid protein marker, Li et al., 2023) and pHELLSGATE-GPAT1-EGFP (mitochondrial membrane marker, Jia et al., 2022) was achieved in *N*. *benthamiana* leaves via *A*. *tumefaciens* (GV3101 strain)-mediated transformation (Norkunas et al., 2018). After 60 h incubation period, confocal imaging analysis was conducted on Zeiss LSM 880 NLO laser scanning confocal microscope systems.

### 2.8 Generation and molecular analysis of GmHIGD3-overexpressing plants

GmHIGD3-overexpressing lines were generated in Col-0 background using pHB-GmHIGD3-mCherry vector and the floral dip method (Clough and Bent, 1998). Putative transgenic lines were selected based on hygromycin resistance and PCR analysis. Three T3 transgenic lines were selected for catalase enzyme activity analysis. For the determination of catalase activities, approximately 100 mg fresh leaves of *Arabidopsis* were homogenized in 1 mL of PBS (pH 7.8, 50 mM PBS). The catalase activity assay was performed using the Catalase Assay Kit (BC0200, Solarbio, Beijing, China) in accordance with the manufacturer’s instructions. Each experiment was performed with three independent replicates.

### 2.9 Tissue expression pattern analysis based on RNA sequencing data

The expression levels of *GmHIGD* genes in seven soybean tissues were obtained from Fragments Per Kilobase per Million (FPKM) values at Phytozome v13 (Goodstein et al., 2012). A heatmap of *GmHIGD* genes was constructed using TBtools to visualize expression levels in different tissues based on the FPKM values. Flower tissue was collected from opened flowers grown in the field during the flowing stage. Root, lateral root, root tip, shoot tip, leaf, and stem tissues were collected from 4-week-old plants grown on B&D medium (Libault et al., 2010). The seed stages were determined based on weight range: S1 < 10 mg; S2, 30–50 mg (storage cells have large central vacuoles); S3, 70–90 mg (storage protein accumulation has begun and vacuole subdivision is occurring); S4, 115–150 mg; S5, 200–250 mg (storage vacuoles are filling); S6, >300 mg (green seeds); S7, >300 mg (yellow seeds); S8, 200–250 mg (fully-mature, yellow and dehydrating seeds); S9 < 150 mg (yellow and fully dehydrated seeds).

### 2.10 Plant materials, growth conditions and treatments

Soybean Williams 82 seeds were germinated on Petri dishes lined with moist filter paper and then transferred to half-strength MS solution. Seedlings were grown in a growth chamber under a 10-hour photoperiod at temperature of 25°C/22°(day/night) and 50% relative humidity. Plants at the vegetative 1 stage were subsequently transferred to half MS solution containing 15% PEG6000 or 150 mM NaCl for 24 hours and 48 hours, respectively. Roots and the first trifoliolate leaves from 5 plants were collected for *GmHIGD* gene expression analysis. Following collection, samples were immediately frozen in liquid nitrogen and stored at -80°for subsequent total RNA isolation.

For hypoxia treatment, 7-day-old soybean seedling were placed in a sealed container (40 cm x 40 cm x 40 cm) equipped with inlet and outlet valves, and were exposed to hypoxia by flushing N_2_ gas (30ml/S) from a nitrogen tank into the container to maintain hypoxia conditions throughout the experiment. Plants were subjected to hypoxia treatment for durations of 2 hours, 4 hours and 8 hours, respectively. Leaves were sampled from 5 different plants for each treatment with three biological replicates utilized to evaluate gene expression patterns.

Nitro blue tetrazolium (NBT, Cat#298-83-9, Solarbio, Beijing, China) staining detected the presence of superoxide. 20-day-old *Arabidopsis* plants was submerged in water for a duration of 12 hours, following which the leaves were soaked in NBT solutions (0.33 mg/ml) for a period of 2 hours. Following this, the leaves underwent decolorization using 95% ethanol in an 80°C-water bath, with the ethanol solution changed every 10 minutes. After complete fading of the green color in the sample, it was examined under a microscope for imaging.

### 2.11 RNA Isolation, cDNA synthesis, and qRT-PCR

The transcript abundance of all *GmHIGD* genes was investigated using qRT-PCR. Total RNA was extracted using an RNApure Plant Kit (DNase I) (CWBIO, Cat: # CW0559, China) according to the manufacturer’s instructions. Approximately 2 μg of total RNA was converted into cDNA using HiScript III RT SuperMix for qPCR (+gDNA wiper) (Vazyme, Cat: # R111-01, China) in a 20 μL reaction volume according to the supplier’s instructions. The Bio-Rad CFX ConnectTM Optics Module Real-Time PCR System (Bio-Rad, USA) and ChamQ Universal SYBR qPCR Master Mix (Vazyme, Cat: # Q711, China) were used to performe quantitative RT-PCR. The *Gmactin11* gene served as a reference gene and specific primers for *HIGD* genes were used for qRT-PCR validation. Gene expression data obtained via qRT-PCR were normalized to the expression of *GmActin* gene and the 2^-△△Ct^ method was employed to calculate the relative expression of *GmHIGD* genes. Each sample was tested in triplicate and three biological replicates were performed. The primers used for qRT-PCR are given in **Supplementary Table S1**.

## 3 Results

### 3.1 Identification and characterization of *GmHIGD* genes

**Figure 1:**
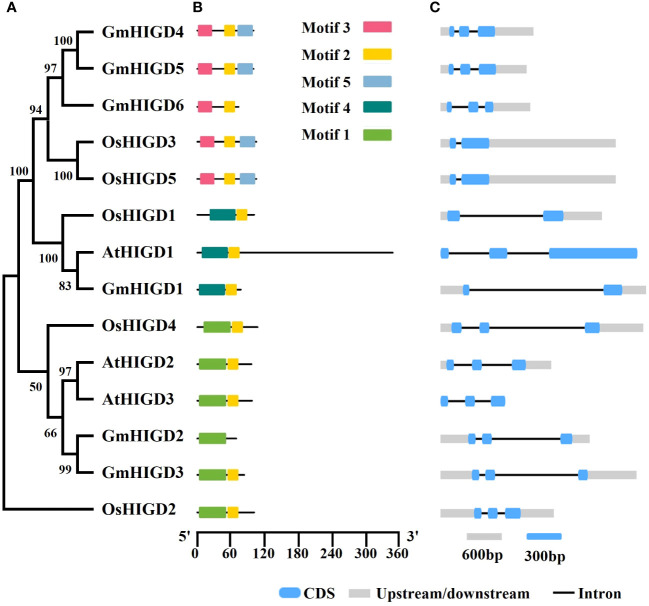
Characterization of soybean GmHIGD genes. **(A)** The phylogenetic tree was constructed with MEGA11 using the neighbor-joining (NJ) method with 1000 bootstrap replicates. The percentage bootstrap scores are indicated on the nodes. **(B)** Motif characterization of HIGD proteins. The motifs are displayed in different colored boxes. **(C)** Gene structure of HIGD genes. Gray boxes indicate untranslated 5′-and 3′-regions; blue boxes indicate exons; black lines indicate introns. Scale bar represents gene length.

HIGD amino acid sequences from rice and *Arabidopsis* were used to identify homologs in the soybean genome (*Glycine max* Wm82.a4.v1). Six HIGD homologs, named GmHIGD1 (chromosome 5), GmHIGD2 (chromosome 10), GmHIGD3 (chromosome 20), GmHIGD4 (chromosome 11), GmHIGD5 (chromosome 18) and GmHIGD6 (chromosome 2), were identified in the Wm82 genome assembly. To further address the evolutionary conservation of GmHIGD proteins, a phylogenetic analysis was performed with three *Arabidopsis* (a model dicot plant), and five rice (a model monocot plant) HIGDs using the neighbor-joining method and bootstrap values from 1,000 replicates. The *HIGD* genes were categorized into three groups based on the tree topology and conserved motifs. *GmHIGD4*, *GmHIGD5*, *GmHIGD6*, *OsHIGD3* and *OsHIGD5* were grouped together; *OsHIGD1*, *AtHIGD1* and *GmHIGD1* were in the same group; while the remaining genes (*AtHIGD2*, *AtHIGD3*, *GmHIGD2*, *GmHIGD3* and *OsHIGD2*) formed another group (**Figures 1A, B**).

The physicochemical properties of the identified GmHIGD protein sequences were evaluated using the ExPASy ProtParam tool (**Table 1**). The amino acid length ranged from 69 to 100 residues, with molecular weight varying from 7952.24 to 11058.91 kDa. The theoretical pI values ranged from 8.95 to 10.38. GmHIGD1, GmHIGD3, and GmHIGD4 were classified as hydrophilic proteins based on their negative grand average of hydropathy (GRAVY index) values, while the remaining three genes were categorized as hydrophobic proteins due to their positive values. Among the identified GmHIGDs, GmHIGD3 and GmHIGD6 exhibited instability index values below 40, suggesting a more stable nature compared to the others with values above 40.

**Table 1:** The physicochemical properties of predicted soybean HIGD proteins.

Gene Name	Gene ID	Number of amino acids	Molecular weight	Theoretical pI	Instability index	Grand average of hydropathicity
GmHIGD1	Glyma.05G035600	77	8444.78	9.98	42.93	-0.014
GmHIGD2	Glyma.10G151300	69	8005.4	10.38	40.13	0.038
GmHIGD3	Glyma.20G236900	83	9154.67	10.01	32.86	-0.082
GmHIGD4	Glyma.11G235000	100	11058.91	8.95	51.65	-0.019
GmHIGD5	Glyma.18G022000	100	11044.93	9.23	43.57	0.05
GmHIGD6	Glyma.02G259700	73	7952.24	10.36	27.97	0.086

Analysis of gene structure using the Gene Structure Display Server (GSDS) revealed that, with few exceptions, *GmHIGD* genes share a conserved genomic structure with two or three exons separated by one or two introns (**Figure 1C**). The structural characteristics of GmHIGD proteins were further plotted based on protein sequence using the MEME motif search tool. The results showed that most of these proteins contained two to three consensus motifs (**Figure 1C**). Soybean HIGD proteins exhibit high conservation with *Arabidopsis* and rice homologs in motif alignment (**Figure 1C**). Closely related genes exhibit similar motif compositions, suggesting functional similarities among HIGDs. Motif 2 is present in all HIGDs among *Arabidopsis*, rice, and soybean, except for GmHIGD2. However, GmHIGD2 only has one conserved motif (motif 1), implying potential functional variations within the *GmHIGD* family. The modeling of *GmHIGDs* tertiary structure were predicated through SWISS-MODEL and verified with SAVES v6.0. As shown in **Supplementary Figure S1**, two α-helix bundles are formed with one β-turn located between the two helixes in all GmHIGDs. The 3D protein structure of HIGDs from *Arabidopsis* and rice were predicated and the results indicated that they all showed the similar structure with that of GmHIGDs (**Supplementary Figure S1**).

### 3.2 Chromosomal location and gene collinearity analysis of GmHIGDs

**Figure 2:**
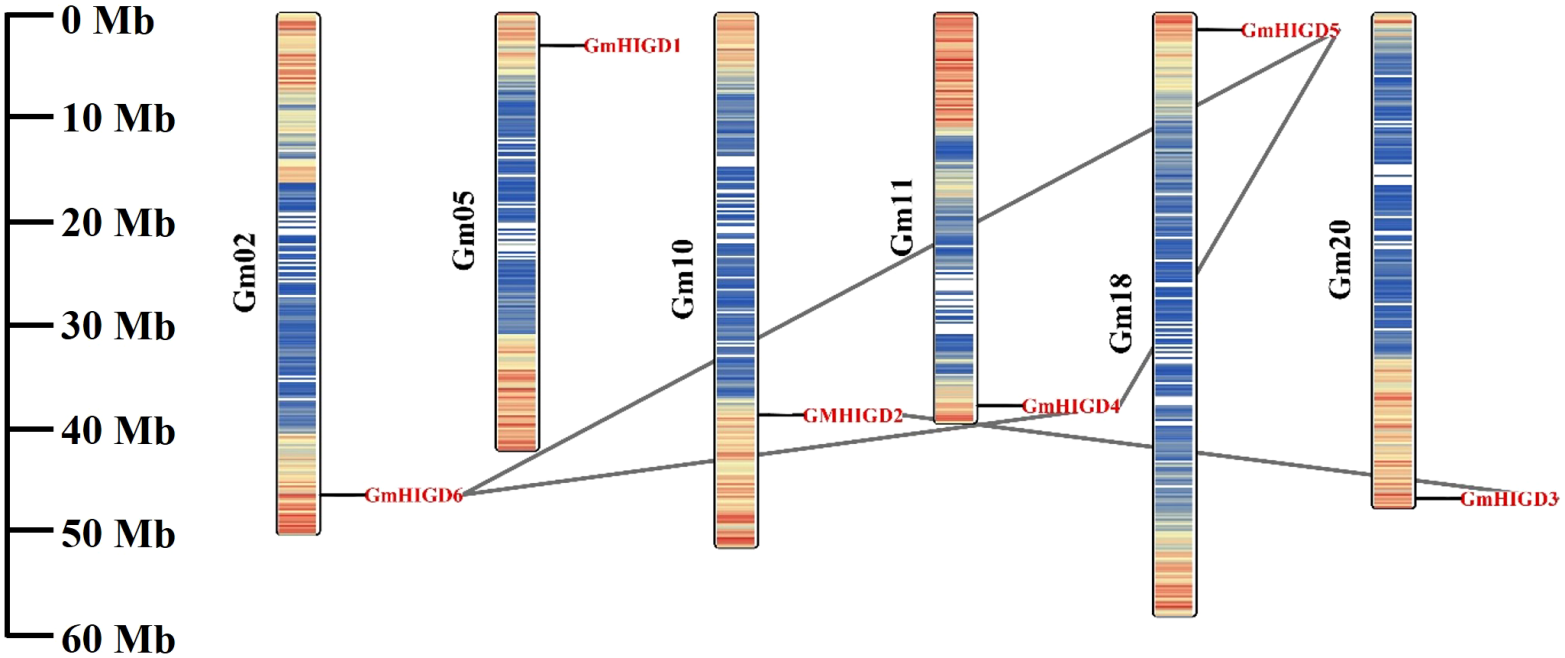
Chromosomal location and gene duplication events of GmHIGD1-6 genes. The scale on the left is in megabases (Mb) and the chromosome numbers are indicated at the left of each bar. The segmental duplication genes have been connected by black straight line.

The chromosomal location of *GmHIGDs* was determined through BLASTN searches in the soybean Wm 82 genome database. As shown in **Figure 2**, all *GmHIGDs* are unevenly distributed on six different chromosomes. Furthermore, gene pairs in the *GmHIGD* family were detected. A total of three gene pairs were detected in the *GmHIGD* gene family, with three genes repeatedly participating in gene duplication events (**Figure 2**).

**Figure 3:**
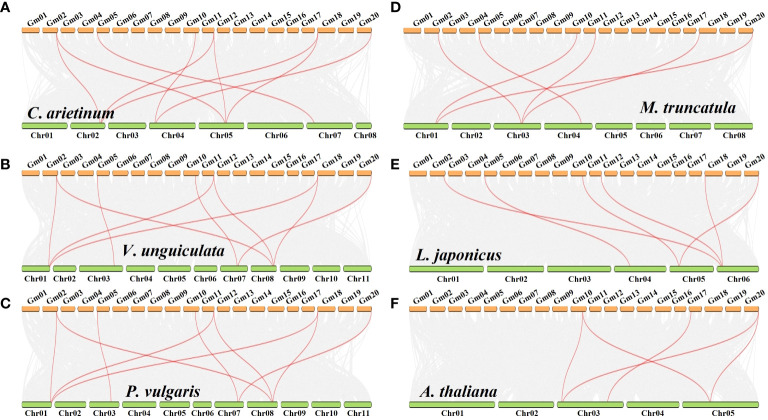
Synteny analysis of GmHIGD with six dicotyledonous plants: *Cicer arietinum* **(A)**, *Vigna unguiculata* **(B)**, *Phaseolus vulgaris* **(C)**, *Medicago truncatula* **(D)**, *Lotus japonicus* **(E)** and *Arabidopsis thaliana* **(F)**. Gray lines represent collinear within soybean and other species, while the red line highlights a one-to-one correspondence of homologous regions of *HIGD* gene pairs. The chromosome number is labeled at the top or bottom of each chromosome.

To explore the evolutionary relationship of *GmHIGD* genes across different species, we constructed nine syntenic soybean maps associated with six dicotyledonous plants *Arabidopsis thaliana*, *Vigna unguiculata*, *Phaseolus vulgaris*, *Lotus japonicus*, *Medicago truncatula*, *Cicer arietinum* (**Figure 3**) and three monocotyledons *Oryza sativa*, *Zea mays* and *Sorghum bicolor* (**Supplementary Figure S2**). Notably, the results indicated a higher homology between soybean and other legume species than that between *Oryza sativa*, *Zea mays* and *Sorghum bicolor*. Specifically, two *Arabidopsis* genes (*AtHIGD2* and *AtHIGD3*) were found to be orthologous with *GmHIGD2* and *GmHIGD3*, respectively. *AtHIGD1* was orthologous with soybean gene (Glyma17g091600), although this gene does not encode a HIGD protein. In addition, only one homologous pair was found between soybean and *Sorghum bicolor*. Three *GmHIGDs* (*GmHIGD4*, *GmHIGD5*, *GmHIGD6*) were found to be orthologous with one *ZmHIGD* gene. Four *GmHIGDs* (*GmHIGD2*, *GmHIGD4*, *GmHIGD5*, *GmHIGD6*) were found to be orthologous with three *OsHIGDs*. These results indicate that the *GmHIGD3* gene may be unique to dicotyledons, whereas *GmHIGD1* is conserved specifically in legumes. The remaining *GmHIGDs* show high conservation during evolution between dicotyledonous and monocotyledonous plants.

### 3.3 Cis-elements analysis of the *GmHIGD* genes promoters and TF identify

**Figure 4:**
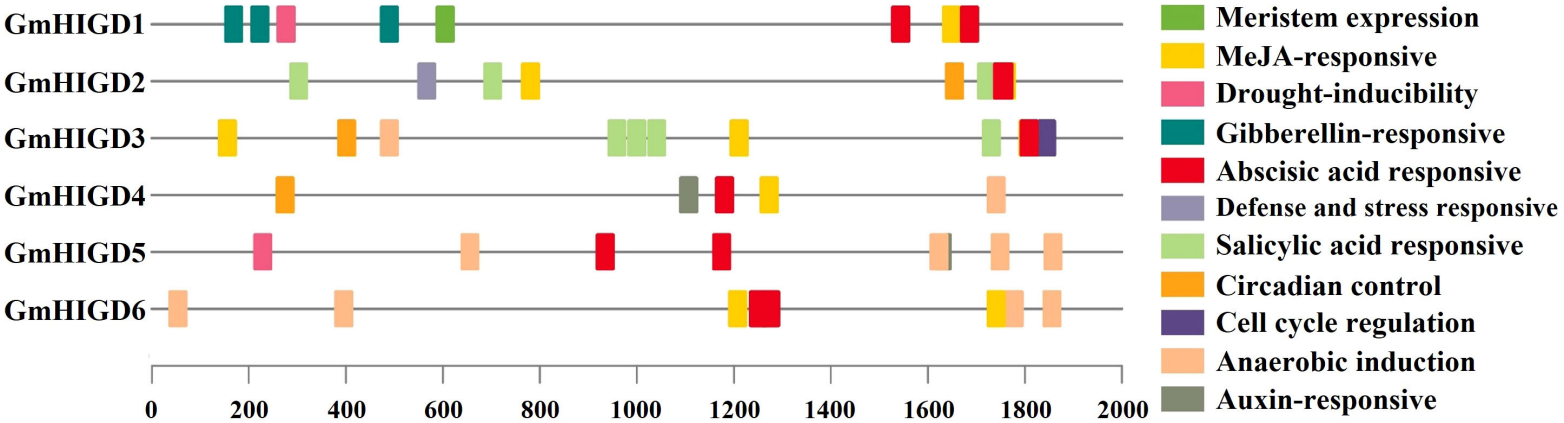
Predicted cis-elements in GmHIGD promoter regions. Promoter sequences (−2000 bp) were analyzed by PlantCARE. The upstream length to the translation starts site can be inferred according to the scale. Legend depicting the color of individual cis elements.

To better understand the roles of *GmHIGDs*, we analyzed their promoter regions. The 2 kb promoter regions were extracted from the soybean genome database and submitted to the plantCARE website (**Figure 4**). As expected, many core promoter elements, such as CAAT-box and TATA-box, were widely distributed in the *GmHIGD* genes. These cis-regulatory element can be categorized into three major groups: abiotic stress response, hormone response and growth and development response. Abiotic stress response elements include anaerobic induction, drought inducibility, defense and stress response elements, like ABRE, MYB, MYC and STRE. Hormone response elements include MeJA, gibberellin, abscisic acid, salicylic acid and auxin response elements. Growth and development response element involve meristem expression, circadian control and cell cycle regulation. These results indicate that the *GmHIGD* gene not only regulates soybean growth and development but also plays an important regulatory role in responding to stress, especially in coping with hypoxia stress.

Furthermore, we predicted transcription factor regulatory networks within the 1 kb promoter sequence using the Plant Transcriptional Regulatory Map. The result revealed that 117 transcription factors (23 families of TFs) might participate in regulating the expression pattern of the *GmHIGDs* family (**Supplementary Table S2**). For instance, the promoter sequence of *GmHIGD1* contained binding sites for ERF transcription factors, while the promoter sequence of *GmHIGD2* had binding sites for bHLH transcription factors. These results suggest that there is a complex regulation network among the different *GmHIGDs*. The identified transcription factors could potentially be valuable for modifying soybean hypoxia response.

### 3.4 *GmHIGD* gene expression analysis across different tissues

**Figure 5:**
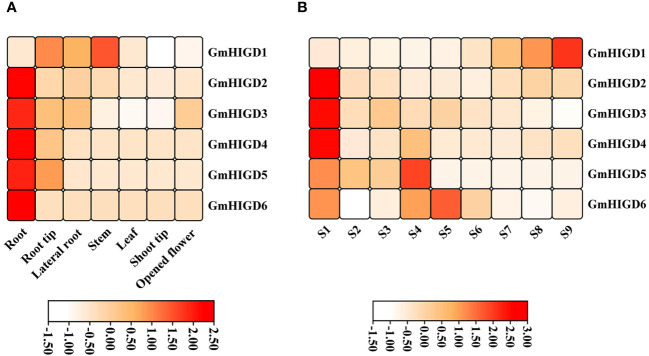
Heatmap showing expression pattern of *GmHIGD* genes across different tissues and seed development stages of soybean. **(A)** different tissues **(B)** different seed development stages. The gene expression was used RNA-seq data derived from mean value of three replicates in each tissue and shown on a scale with z-scaling by row.

To characterize the expression patterns of individual *GmHIGDs* at different stages, we used publicly available RNA-seq data for *G. max*. The RPKM values for seed development and vegetative growth tissues were plotted in a hierarchical heatmap (**Figure 5**). *GmHIGD1*, *GmHIGD2* and *GmHIGD3* were distributed across different tissues, while *GmHIGD2* and *GmHIGD3* had relatively higher levels in the root. On the other hand, *GmHIGD4*, *GmHIGD5* and *GmHIGD6* exhibited greatly expression in root tissues, with *GmHIGD4* and *GmHIGD5* showing elevated expression in the root tip (**Figure 5A**). Notably, *GmHIGD2* and *GmHIGD6* exhibited root-specific expression patterns, suggesting potential specific roles in root development.

At different seed development stages of soybean, *GmHIGD2*, *GmHIGD3* and *GmHIGD4* showed very high expression levels in the early stage of seed development (S1). As for the S4-S5 seed development stage, the expression levels of *GmHIGD5* and *GmHIGD6* were higher than those in other stages; *GmHIGD1* displayed significant expression only in S7-S9 during the late stage of seed development (**Figure 5B**). These results indicate that different *GmHIGD* genes may play distinct functions at different stages of seed development.

### 3.5 Expression patterns of *GmHIGD* genes under flooding and hypoxia stresses

**Figure 6:**
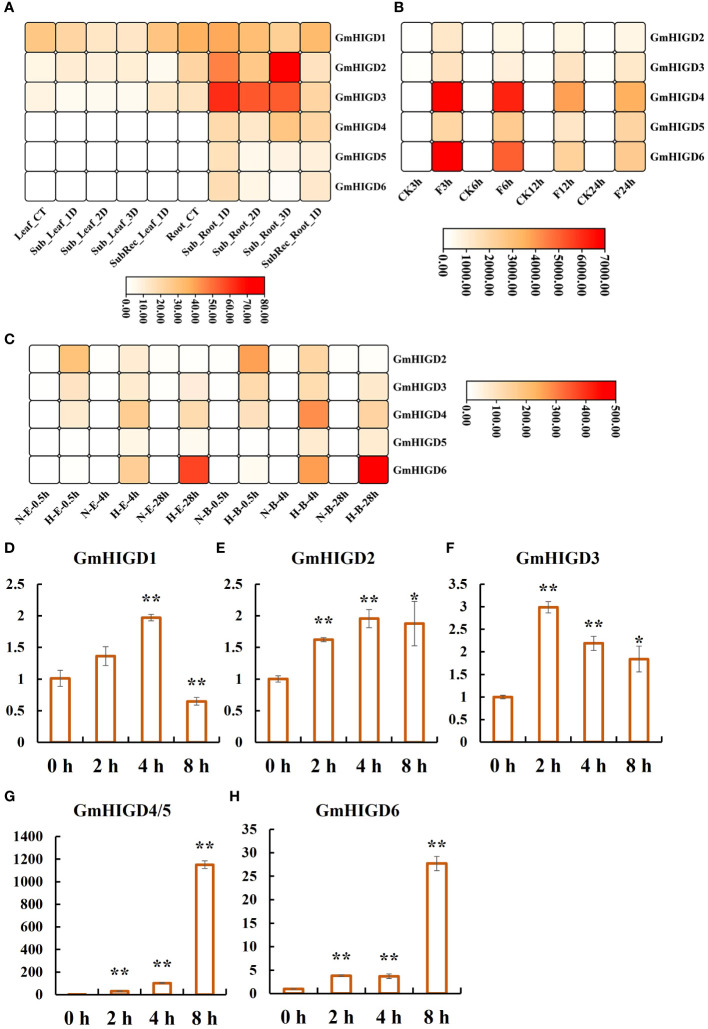
Expression patterns of *GmHIGDs* genes under waterlogging and hypoxic stress. **(A)**. Expression patterns of *GmHIGDs* genes in Williams 82 after 1, 2, and 3 days of flooding and 1 day of recovery after 3 days of flooding, Leaf: leaf tissue, Root: root, CT: control, Sub: flooding, Sub Rec: recovery after 3 days of flooding; **(B)**. Expression patterns of *GmHIGDs* gene in roots of Qihuang 34 under water flooding for 3, 6, 12, and 24 hours, CK: control, F: water flooding; **(C)**. Expression pattern of *GmHIGDs* gene in roots of Embrapa 45 and BR 4 under hypoxia for 0.5h, 4h, and 28h, N: non hypoxia, H: hypoxia; E: Embrapa 45, B: BR 4. **(D–H)** Expression analysis of *GmHIGD* genes by quantitative real-time PCR in soybean seedlings under hypoxia treatment for 0h, 2h, 4h and 8h, respectively. *, *p* < 0.05; **, *p* < 0.01.

To evaluate the expression profiling of *GmHIGD* genes under flooding and hypoxia conditions, we used RNA-seq data from three studies. According to the results reported by Tamang et al. (2021), no differentially expression pattern was found in leaf tissues after 1-3 days submergence or 1 day of recovery following 3 days of submergence. However, all *GmHIGD* genes showed high expression levels in root tissues under treatment. In particular, *GmHIGD2* and *GmHIGD3* were highly induced after 2- or 3-days submergence treatment (**Figure 6A**). Lin et al. (2019) reported all *GmHIGD* genes showed relatively high expression levels under submergence treatment in root tissue of the Qihuang34 variety. Especially, *GmHIGD4* and *GmHIGD6* were highly induced after 3 h or 6 h compared to non-treatment (**Figure 6B**). In addition, we further investigated the expression of *GmHIGD* genes in root tissue of flood-tolerant Embrapa 45 and flood-sensitive BR 4 soybean cultivars under hypoxic stress (Nakayama et al., 2017). As shown in **Figure 6C**, the expression of five *GmHIGD* genes was enhanced in both cultivars under hypoxia induction at different time points. These results showed that all *GmHIGD* genes are specifically responsive to submergence in root tissues, indicating a positive role of the *GmHIGD* family in the soybean’s response to submergence processes.

We further analyzed the expression of six *GmHIGD* genes in Wm82 under hypoxia conditions with N_2_ treatment using the qPCR method (**Figures 6D–H**). Due to high homology in gene sequences between *GmHIGD4* and *GmHIGD5*, the designed primers amplified both genes simultaneously in this study. The results showed that the transcripts of *GmHIGD4*/*5* were largely accumulated (30 to 1100-fold) after 2-8 hours of hypoxia treatment. *GmHIGD6* was induced over 20-fold after 8 hours of hypoxia treatment in soybean seedlings (**Figures 6D–H**). These results suggest that the expression of the *GmHIGD* gene family is regulated by flooding and hypoxic stress.

### 3.6 Expression patterns of *GmHIGD* genes under drought and salt condition

**Figure 7:**
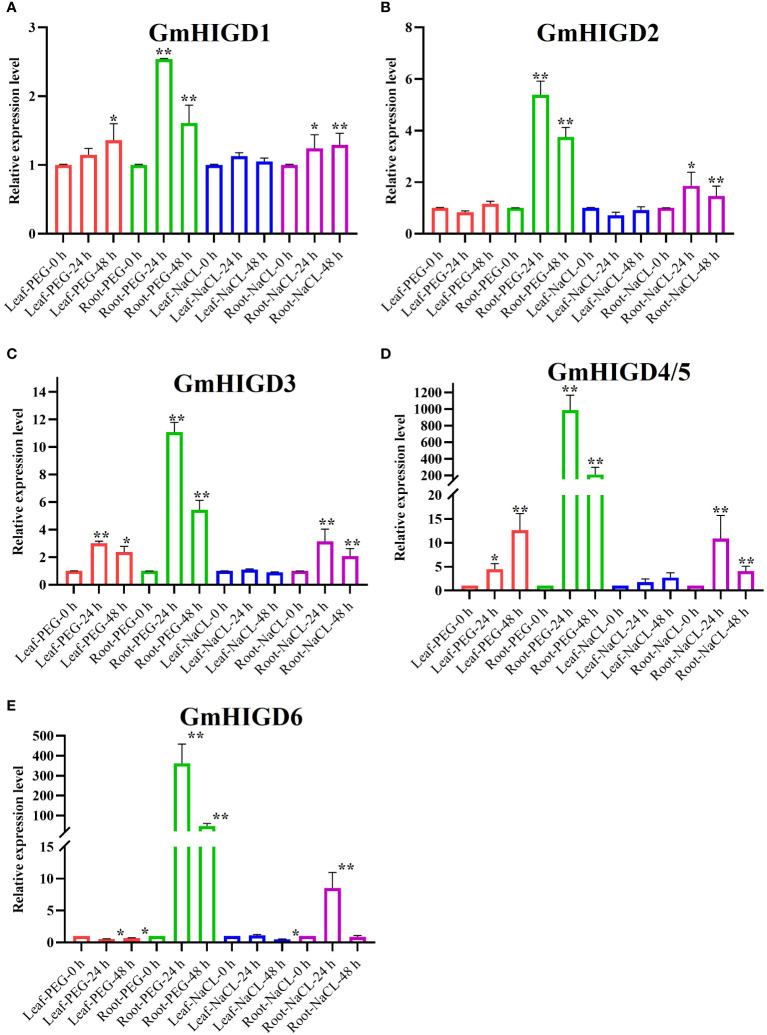
Expression profiles of *HIGD* genes, measured by real-time qRT-PCR in Williams 82 ecotype as compared with PEG and NaCl treatments. **(A)** The relative expression levels of *GmHIGD1* gene. **(B)** The relative expression levels of *GmHIGD2* gene. **(C)** The relative expression levels of *GmHIGD3* gene. **(D)** The relative expression levels of *GmHIGD4* gene. **(E)** The relative expression levels of *GmHIGD5* gene. The relative gene expression levels were calculated relative to 0 h and using 2^−△△CT^ method. The data shown are the mean values SE of three replicates. * and ** indicates that there are significant differences at 5%, 1% level respectively relative to controls.

To understand the effects of drought and salt stress on *GmHIGD* gene expression, we detected the transcript abundance of *GmHIGDs* in soybean roots and leaves by qPCR after 24 hours and 48 hours of PEG or NaCl treatment, respectively. As shown in **Figure 7**, *GmHIGD1*, *GmHIGD3* and *GmHIGD4*/*5* genes showed significantly upregulaton in both leaves and roots after PEG treatment (**Figures 7A, C, D**). *GmHIGD2* transcript levels were up-regulated in root tissues under PEG or NaCl treatment (**Figure 7B**). The expression levels of *GmHIGD4/5* and *GmHIGD6* was significantly and highly increased in root tissues under PEG or NaCl treatments, especially under PEG treatment (**Figures 7D, E**). In contrast, the expression level of *GmHIGD6* in leaves was downregulated, with no significant changes found in other *GmHIGD* genes after NaCl treatment in leaves. These results suggest that *GmHIGDs* may participate in the response and resistance of soybean to drought and NaCl stress.

### 3.7 Function prediction of *HIGD* genes

To further explore the function of *GmHIGD*, six *GmHIGDs* genes were selected as ‘guide genes’ to identify co-expressed genes using expression data from the Phytozome database with a Pearson’s correlation coefficient (PCC) threshold of 0.7 (Aoki et al., 2007). Meanwhile, the STRING database was used to build an interaction network between GmHIGDs proteins and other soybean proteins, focusing on genes with a trusted value (medium confidence, 0.4). Finally, 319 genes exhibiting closely correlated expression patterns to or interaction with GmHIGDs were identified. GO annotation analysis of these 319 gene revealed that their involvement in a range of molecular functions, with enriched annotations predominantly in oxidoreductase activity, dioxygenase activity, cation transmembrane transporter activity, transcription factor or translation factor activity (**Supplementary Table S3**). In addition, the KEGG pathway analysis indicated enrichment in amino acid metabolism, carbohydrate metabolism, energy metabolism and peroxisome pathways (**Supplementary Figure S3**).

### 3.8 Characterization of the subcellular location and function of GmHIGD3

**Figure 8:**
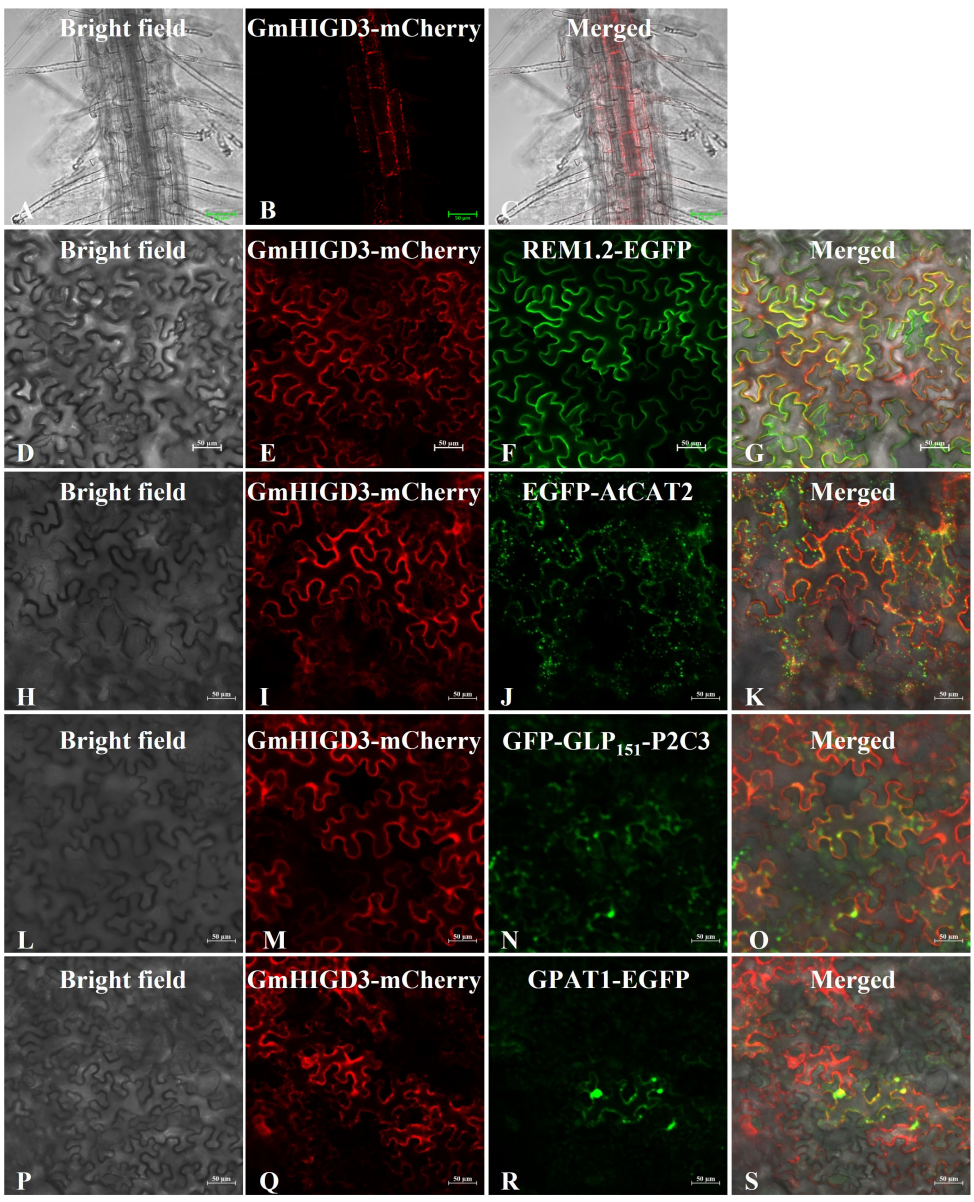
Subcellular localization of GmHIGD3 by confocal laser scanning microscopy. **(A–C)** Subcellular localization of GmHIGD3-mCherry in *Arabidopsis* root. Epifluorescence images of tobacco leaf cells infiltrated with Agrobacterium harboring the different fusion constructs; **(D–G)** GmHIGD3-mCherry and REM1.2-EGFP; **(H–K)** GmHIGD3-mCherry and AtCAT2-EGFP; **(L–O)** GmHIGD3-mCherry and GLP_151_-P2P3-GFP; **(P–S)** GmHIGD3-mCherry and GPAT1-EGFP.

The subcellular localization of GmHIGDs predicated by different online tools may be located in diverse organelles, such as mitochondria, chloroplast, peroxisome, nucleus or vacuole (**Supplementary Table S4**). We further validate the subcellular localization of GmHIGD3 *in vivo* by using overexpressed transgenic *Arabidopsis* seedlings. Fluorescence analysis of GmHIGD3-mCherry expression revealed small punctate structures in root cells, which appeared to be localized to mitochondria (**Figures 8A–C**). The localization of GmHIGD3-mCherry was further confirmed through co-localization experiments with known organelle markers in tobacco leaf infiltration studies. As shown in **Figure 8D–O**, there was a partial overlap in the colocalization of GmHIGD3-mCherry fusion protein and REM1.2 (membrane localized protein), or AtCAT2 (peroxisomal protein), or GLP_151_-P2P3 (plastid localized protein). By contrast, significant co-localization was observed with the mitochondrial marker GPAT1-EGFP when co-expressed (**Figures 8P–S**). These results strongly suggest that GmHIGD3 is predominantly localized in mitochondria.

**Figure 9:**
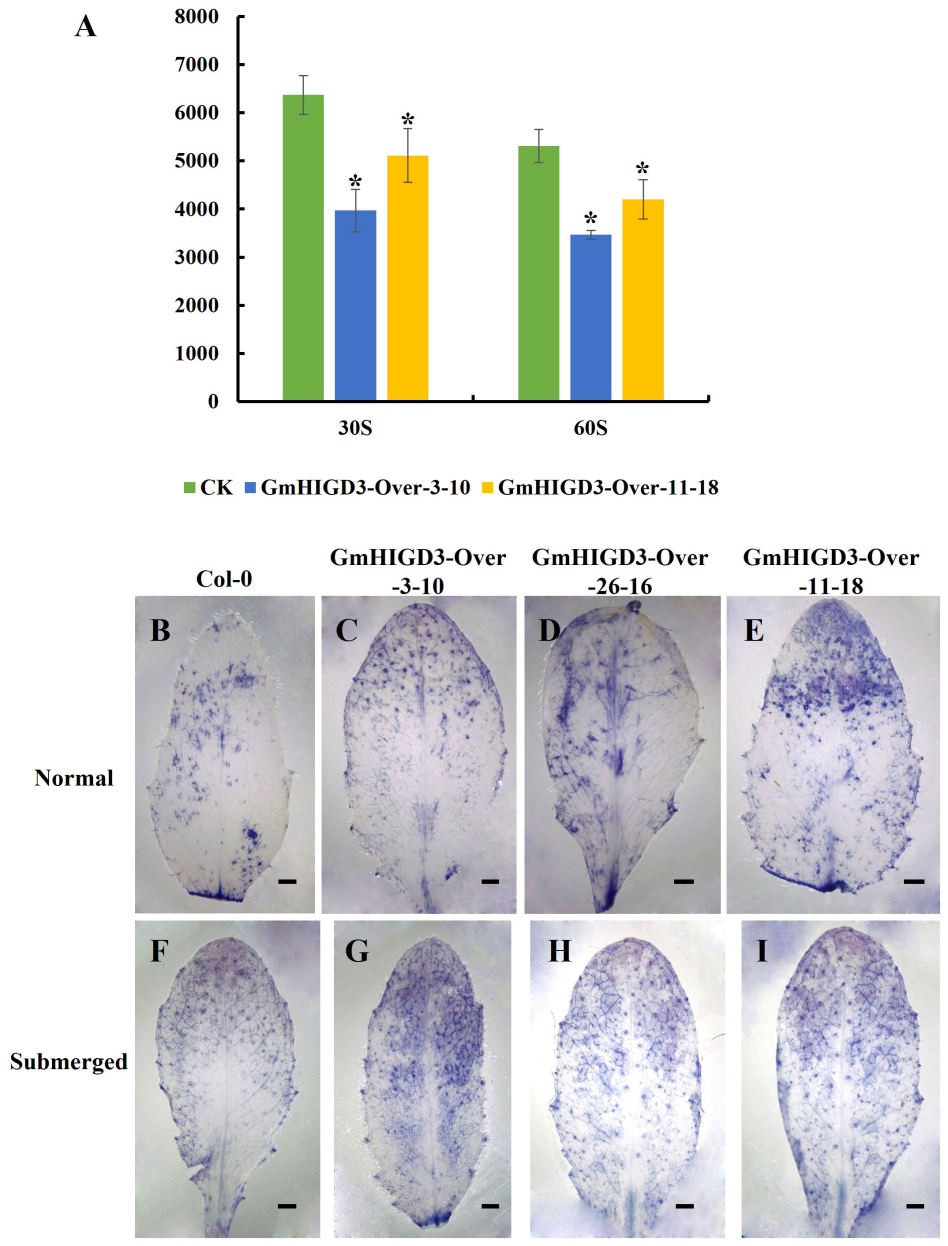
The evaluation of catalase activity and ROS content in GmHIGD3 overexpressing transgenic lines and wild type. **(A)** The catalase activity was inhibited in GmHIGD3 overexpressing compared with wild type. * Indicates a significant difference at the 1% level compared to the non-transgenic control. **(B–I)** Superoxide contents were detected by 0.33 mg/mL of NBT staining. Experiments were repeated three times with similar results. Bars represent 1 mm.

The transgenic *Arabidopsis* plants overexpressing *GmHIGD3* were generated to investigate its function further. Since GmHIGD3 is primarily located in mitochondria and its co-expressed genes are mainly involved in oxidoreductase activity, the catalase enzyme activity was compared between the transgenic lines and wide type. As shown in **Figure 9A**, the catalase activity in the overexpressed *GmHIGD3* transgenic lines is significantly lower than that in the wild type, suggesting that GmHIGD3 plays an important role in regulating oxidoreductase activity. Furthermore, the accumulation of superoxide was detected using NBT staining. Increased staining was observed in the GmHIGD3-overexpressing transgenic lines compared with wild type under normal and submerged conditions, whereas no significant difference was observed between normal and submerged conditions in GmHIGD3-overexpressing transgenic lines (**Figures 9B–I**). This result indicates that the downregulated catalase enzyme activity in the GmHIGD3-overexpressing transgenic lines leads to the accumulation of reactive oxygen species (ROS).

## 4 Discussion

A comparison of all known mammalian and plant *HIGD* genes reveals that the number of HIGD protein is relatively conserved, typically ranging from 3 to 5. As such, the *HIGD* family is not a large or plant-specific group. Members of the *HIGD* family has not been extensively studied at a genome-wide level in plants. In this study, we focuses on the identification and characterization of six homologous soybean *HIGD* genes. Furthermore, more predicted orthologous relationships were found in dicots compared to monocots, indicating that their functions may have diverged throughout evolution. Conserved domain and motifs analysis indicated that GmHIGD1 shares conserved domains with AtHIGD1. Proteins within the same subgroup exhibit similar motifs, which might be related to their specific functions.

The expression of *HIGD2A* is dependent on oxygen levels, glucose concentration, and cell cycle progression in human and animals. While the potential roles of the *HIGD* gene family in response to biotic and abiotic stresses are recognized, there is limited research on these genes in plant species. In rice and *Arabidopsis*, the expression patterns of *HIGD* genes change under stress conditions. Hwang and Choi (2016) studied the expression patterns of *OsHIGDs* in rice and found that *OsHIGD2* exhibited a significant response to submergence and hypoxia, with a slight response to ethylene. *OsHIGD3* and *OsHIGD5* were slightly induced by submergence and hypoxia. Except for *OsHIGD2*, no other *OsHIGDs* displayed differential expression in response to ethylene, suggesting that *OsHIGD2* might be the most active member of the *OsHIGD* gene family. Similarly, *AtHIGD1* was upregulated at 8, 16 and 24 hours after hypoxia treatment (Hwang et al., 2017). In our current study, we found that all *GmHIGDs* respond to abiotic stress, especially to hypoxia. Among them, the expression level of *GmHIGD4/5* sharply increased after 8 hours of hypoxia induction, followed by *GmHIGD6.* Furthermore, we examined the expression of *GmHIGD* gene family in root and leaf tissues at different time points after PEG treatment. The expression levels of *GmHIGD4/5* and *GmHIGD6* in roots were significantly higher than in the control under PEG stress conditions, suggesting that *GmHIGD4/5* and *GmHIGD6* might be the most active members of the *GmHIGD* gene family in response to abiotic stress. At the same time, *GmHIGDs* were highly expressed in soybean roots. Given that roots play an important role in water and nutrients absorption, the *GmHIGD* gene family may play an vital role in the development of soybean root systems under stress conditions.

To clarify the functional positions of GmHIGDs in plant cells, an online tool was initially used to predict that GmHIGD2 and GmHIGD3 are located in mitochondria. Furthermore, our finding indicates that GmHIGD2 and GmHIGD3 are not only located in mitochondria, but also in the cell membrane by tobacco epidermal cell experiment. It has been reported that HIGD1A and HIGD2A were embedded in the mitochondrial inner membrane in mammals (Strogolova et al., 2012). In addition to their mitochondrial location, studies have shown that the localization of HIGD proteins may change in response to certain stressors. For example, HIGD1A has been found to relocate to the nucleus during apoptosis triggered by severe metabolic or DNA damage stressors (Ameri et al., 2013 & Ameri et al., 2015), and HIGD2A has been observed in the nucleus even under physiological conditions (Salazar et al., 2019). López et al. (2018) also reported that HIGD1A migrates from the cytoplasmic pool to the nucleus in conditions like ischemic heart disease, cancer, and ischemic encephalopathy. Further experiments are needed to investigate whether the localization of GmHIGD proteins changes in response to environmental stimuli such as hypoxia or drought.

Recent studies in mammals have provided convincing evidence of a strong correlation between *HIGD* and oxygen consumption, ROS production, and tumor growth (Shahrzad et al., 2007; Ameri et al., 2015; Timón-Gómez et al., 2020). While there have been numerous studies on the function of the *HIIGD* gene family in mammals, research on its role in plants is limited. Currently, only the function of AtHIGD1 in *Arabidopsis thaliana* has been preliminarily studied, and it participates in the plant’s hypoxia response. With global climate change leading to more frequent flooding and subsequent hypoxia in plant, decreased oxygen levels can severely affect mitochondrial energy generation, causing intense damage (Nakamura and Noguchi, 2020; León et al., 2021; Barreto et al., 2022; Jethva et al., 2022). In hypoxic conditions, cell acidification and ROS accumulation negatively affect overall plant growth. The transition of hypoxia–reoxygenation responses can cause excess generation of ROS, potentially leading to cellular damage (Hebelstrup and Møller, 2015; Turkan, 2018). At the same time, the degradation of ROS via activation of antioxidant mechanism is equally important to prevent cell damage and maintain cellular homeostasis (Lee et al., 2020; Pucciariello and Perata, 2021). Moreover, due to enhanced denitrification under anaerobic conditions, flooding reduces the availability of soil nitrogen, leading to crop loss (Sjøgaard et al., 2018; Yu et al., 2020).

Co-expression analysis is usually used to identify functional factors participating in specific biological processes (Fu and Xue, 2010). In this study, we found that *GmHIGD* genes may be associated with oxidoreductase activity, dioxygenase activity, and cation transmembrane transporter activity based on co-expression analysis. This suggests taht *GmHIGD* genes could contribute to oxidation-reduction processes, cellular nitrogen compound biosynthetic processes, organic cyclic compound biosynthetic processes and heterocycle biosynthetic processes. Additional functional characterization of GmHIGDs and exploration of the transcriptional network related to hypoxia response need to be carried out.

## 5 Conclusions

In soybean, six *HIGD* genes were identified and their protein physicochemical properties were investigated. Their chromosome location, gene structure, promoter cis-elements, conserved motif, evolutionary relationships and subcellular location were analyzed. The results indicated that *GmHIGD* genes are highly conserved, with five of them exhibiting higher expression levels in root tissue compared to other tissues. Additionally, all *GmHIGDs* responded to hypoxia, flooding, drought and salt stresses at different time points. Functional analysis of *GmHIGD3* illustrated that it is localized to mitochondria and decreased the catalase activity when over-expressed. Co-expression gene enrichment analysis illustrated the biological processes involved in somatic embryogenesis. These compressive analysis of the soybean *HIGD* gene family provides useful insights for further study of the function of *GmHIGD* genes in hypoxia tolerance.

## Data availability statement

The original contributions presented in the study are included in the article/**Supplementary Material**. Further inquiries can be directed to the corresponding author.

## Ethics statement

The plant material used in this study was Williams 82, which was planted in the growth chamber of Yangzhou University, Yangzhou, China, and no permits are required for the collection of plant samples. This study did not require ethical approval or consent as did not involve any endangered or protected species.

## Author contributions

XG: Data curation, Methodology, Writing – original draft. LD: Formal analysis, Investigation, Writing – original draft. TZ: Investigation, Writing – original draft. CY: Investigation, Validation, Writing – original draft. JZ: Investigation, Methodology, Writing – original draft. BG: Software, Supervision, Writing – review & editing. HC: Supervision, Writing – original draft. QZ: Supervision, Writing – review & editing. LS: Funding acquisition, Resources, Visualization, Writing – original draft, Writing – review & editing.

## Supplementary material

Supplementary Figure 1 Predicted three-dimensional structures of the GmHIGD, AtHIGD and OsHIGD protein sequences. Models were constructed using SWISS-MODEL.

Supplementary Figure 2 Synteny analysis of GmHIGD with three monocotyledonous species (*O*. sativa, *Z*. *mays* and *S*. *bicolor*) by MCscan. Gray lines represent collinear within soybean and other species, while the red line highlights a one-to-one correspondence of homologous regions of HIGD gene pairs. The chromosome number is labeled at the top or bottom of each chromosome.

Supplementary Figure 3 Enrichment analysis of co-expressed genes of GmHIGDs.
