# Supplementary figures and images for: Genome-wide analysis of soybean hypoxia inducible gene domain containing genes: a functional investigation of GmHIGD3
Source: Front Plant Sci. 2024 Jul 1;15:1403841. doi: 10.3389/fpls.2024.1403841 (PMC11246964; doi:10.3389/fpls.2024.1403841)

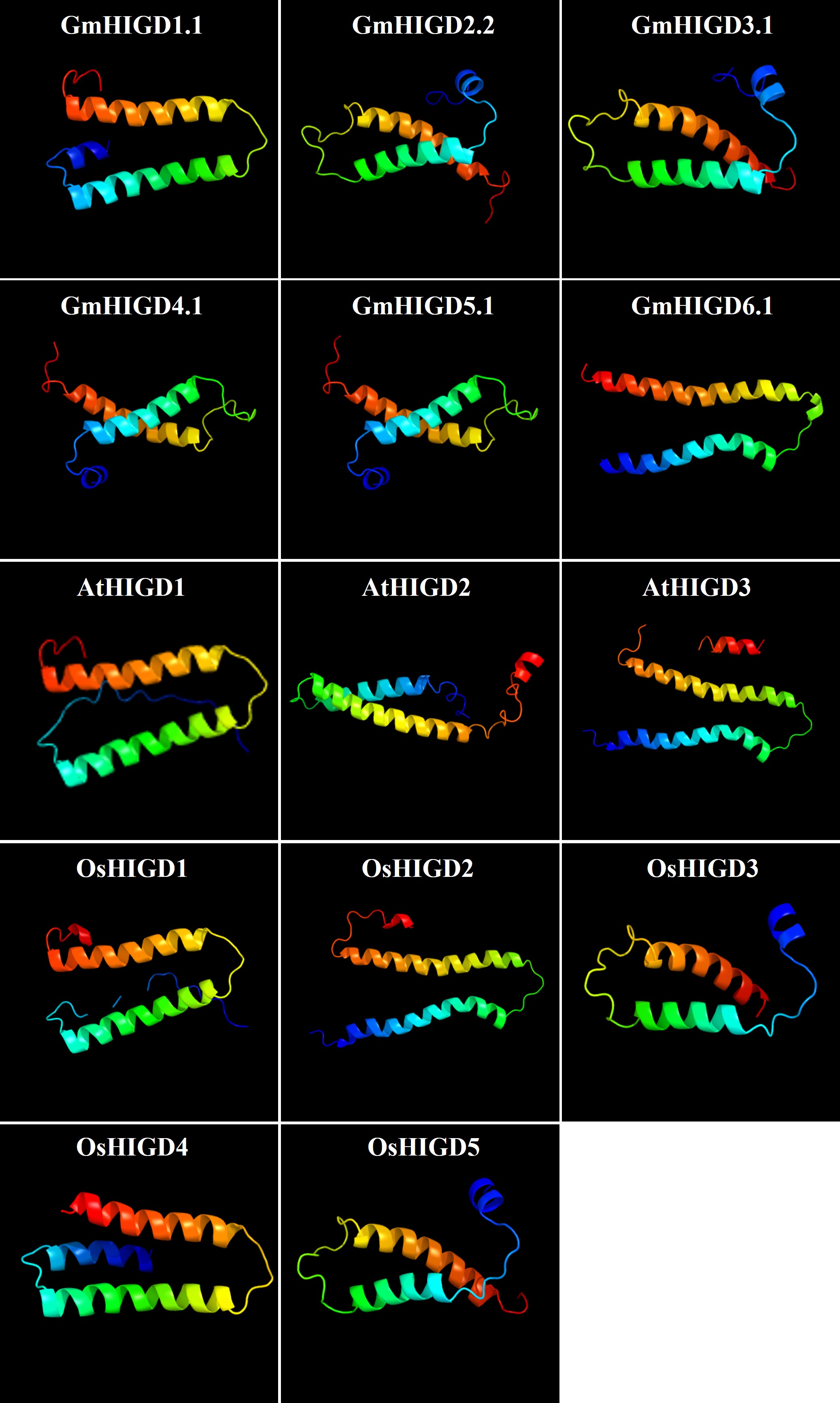

Supplement: Supplementary Figure 1 — Predicted three-dimensional structures of the GmHIGD, AtHIGD and OsHIGD protein sequences. Models were constructed using SWISS-MODEL. [file Image_1.jpg]

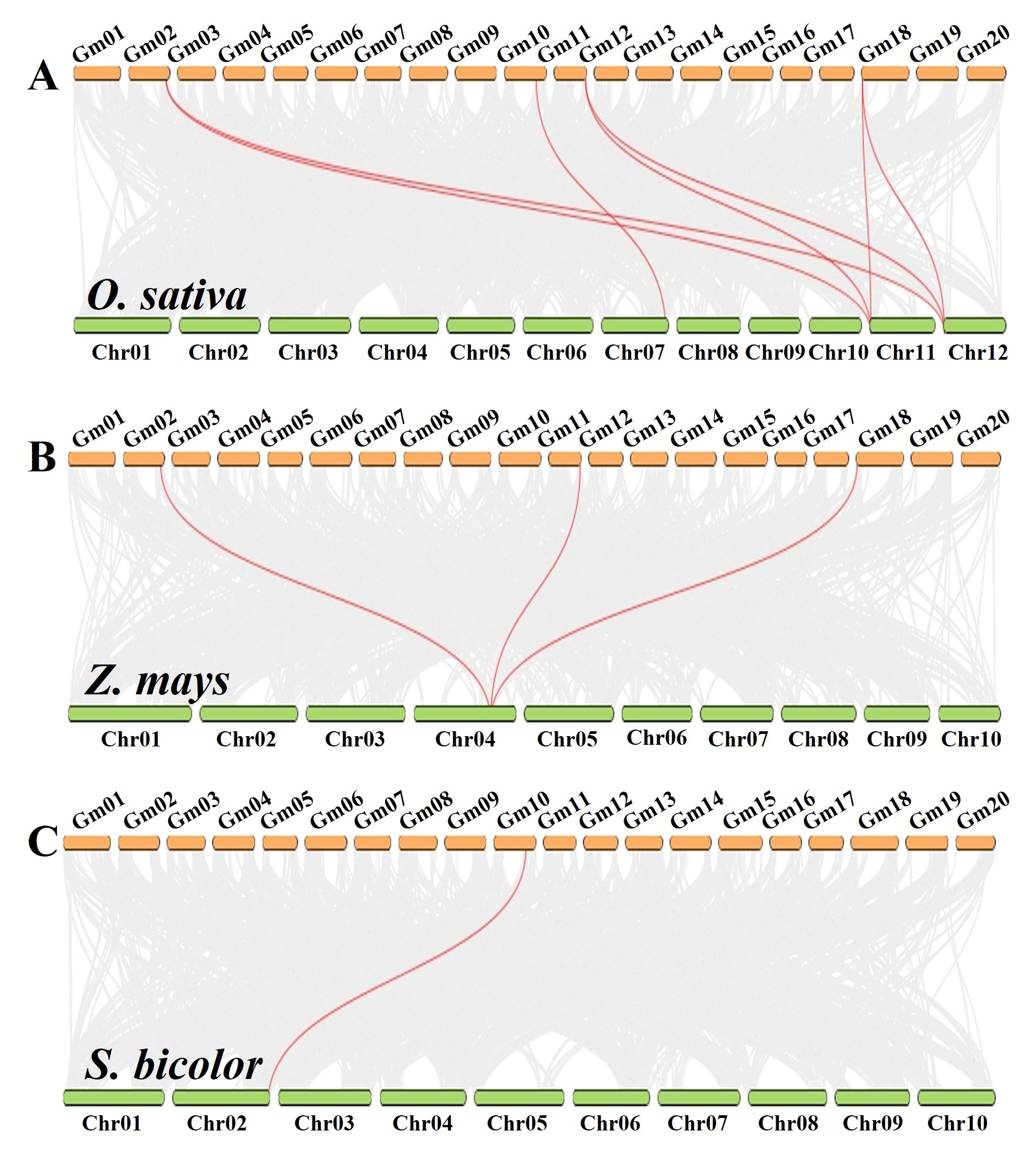

Supplement: Supplementary Figure 2 — Synteny analysis of GmHIGD with three monocotyledonous species (O. sativa, Z. mays and S. bicolor) by MCscan. Gray lines represent collinear within soybean and other species, while the red line highlights a one-to-one correspondence of homologous regions of HIGD gene pairs. The chromosome number is labeled at the top or bottom of each chromosome. [file Image_2.jpg]

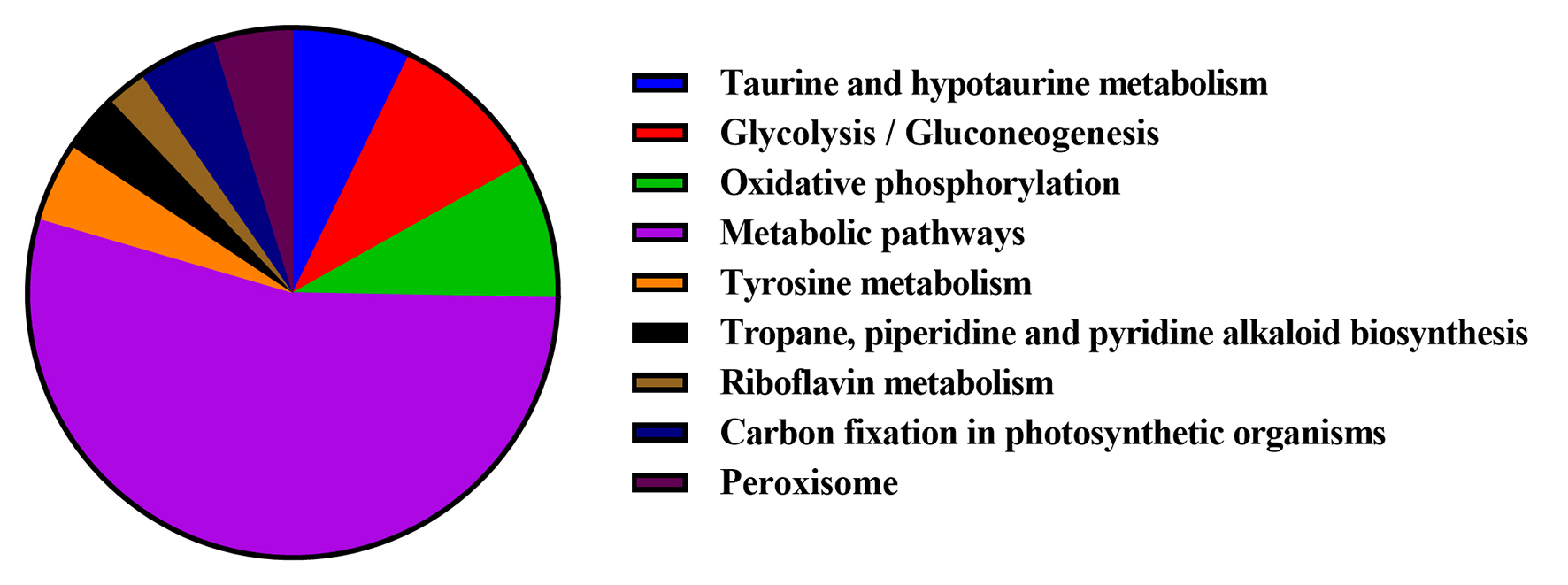

Supplement: Supplementary Figure 3 — Enrichment analysis of co-expressed genes of GmHIGDs. [file Image_3.jpg]
