# Supplementary material for: Genome-wide analysis of soybean hypoxia inducible gene domain containing genes: a functional investigation of GmHIGD3
Source: Front Plant Sci. 2024 Jul 1;15:1403841. doi: 10.3389/fpls.2024.1403841 (PMC11246964; doi:10.3389/fpls.2024.1403841)
Supplement: Supplementary file 4 [file DataSheet_1.docx]

>AtHIGD1

MSSVEPDMEDLFQEKKRVRNPLVPLGALMTAGVLTAGLISFRRGNSQLGQVLMRARVVVQ

GATVALMVGTGYYYGDNPWKKLLLSEIHETEALSPKSSSAATLTLMNQKDPSSSSIVSVL

CLVISGLALIIVFLGVLYLIFKFLRKSSTLFPIPHFNYNPDLFSFSSPQLQHLFFLHDSG

LDQTAIDALPVFLYGNVTISLEQPFDCAVCLNEFSDTDKLRLLPVCSHAFHLHCIDTWLL

SNSTCPLCRRSLSTSNVCYNHSETLVAPLSGHQQVDDGKASLAKRVFSVRLGRFKSTNES

QSQRHDVKDEIGVRMPRRCYSMGTQQYLVCDQDFVVALSSSPREGNIGR*

>AtHIGD2

MAEPKTKVAEIREWIIEHKLRTVGCLWLSGISGSIAYNWSKPAMKTSVRIIHARLHAQAL

TLAALAGAAAVEYYDHKSGATDRIPKFLKPDNLNKD*

>AtHIGD3

MVESKTKFEEIRKWVSDHKLRTVGCLWLSGITGSIAYNWSQPAMKTSVKIIHARLHAQAL

TLAALAGAAVVEYYDHKTEATNRYPKFLPPENLSHKD*

>OsHIGD1

MEKSSTAAGGRSSTPPPPMYTDEFALEGKKPVKNPFVPIGALVTAGVLTAGLISFRYGNS

KLGQKLMRARVVAQGATVALMIGSAYYYGDQIKLFKKGSSP*

>OsHIGD2

MAEEKSTMQSMREWVVDHKLRAVGTLWLTGVASSIAYNWSRPGMKTSVKIIHARLHAQAL

TLAALAGSALVEYYDHRSGSGSRVHQYAKQFITPESNPQKE*

>OsHIGD3

MERLSSSVQSWVEEHKLASIGGLWATAVGASVAYGRRKTPQMRLIHARLHAQALTLAVLG

GAALAHHYYNPSSNTNNSSSLDYDFYSQLPPATTDDGQENERWSW*

>OsHIGD4

MGEEAAKQMAEAPGKIESMRKWVIDHKLRAVGCLWLTGISSSIAYNWSRPNMKTSVKIIH

ARLHAQALTLAALVGSAMVEYYDAKYGTSGPKVDKYTSQYLAHSHKD*

>OsHIGD5

MERLSSSVQSWVEEHKLASIGGLWATAVGASVAYGRRKTPQMRLIHARLHAQALTLAVLG

GAALAHHYYNPSSKTNNSSSLDYDFYSQLPPATTDDGQENERWSW*

>GmHIGD1

MAEDYLQEEKKRVRNPFVPIGALVTAGVLTAGLISFRQGNSQLGQKLMRARVVVQGATVA

LMVGTAFYYGENPWRSS*

>GmHIGD2

MSEAKTQIESIRKWVVEHKLRTVGCLWLSGISGSIAYNWSRPNMKTSVKIIHARLHAQALTLGALAGAALVEYYDRNAGAKASKEILDNK*

>GmHIGD6

MDAIQLWVSKHKLATVGGLWASGIGASLVAYSRTRSPMKPSLRLIHARLHAQALTLAVLS

GAAAYRYYENRAD*

>GmHIGD3

MSEAKTQIESIRKWVVEHKLRTVGCLWLSGITGSIAYNWSRPNMKTSVKIIHARLHAQAL

TLGALAGAALVEYYDRKTGAKAS*

>GmHIGD4

MEALQSWVSKHKLASIGALWASGIGATLVAYSCKKSPMKPSLRLIHARMHAQALTLAVLS

GAAAYHYYEKRDVQPKPEADYIIPAPNVTQMVEYELQCPF*

>GmHIGD5

METVQSWVSKHKLASIGALWASGIGATLVAYSCKKSPMKPSLRLIHARMHAQALTLAVLS

GAAVYHFYEKRDVQPKPVADYTIPAPNVTQMVEYELQCPF*
